# Over a Century of Study and Still Misunderstood: Recognizing the Spectrum of Acute and Chronic Wernicke–Korsakoff Syndrome

**DOI:** 10.3390/jcm12216880

**Published:** 2023-10-31

**Authors:** Simon J. Scalzo, Stephen C. Bowden

**Affiliations:** 1 Melbourne School of Psychological Sciences, Redmond Barry Building, University of Melbourne, Parkville, VIC 3010, Australia; sbowden@unimelb.edu.au; 2 Centre for Clinical Neuroscience & Neurological Research, St Vincent’s Hospital Melbourne, Fitzroy, VIC 3065, Australia

**Keywords:** Wernicke–Korsakoff syndrome, alcohol-related disorders, memory disorders, neuropsychology, alcohol-related dementia

## Abstract

The aim of this study was to objectively evaluate the hypothesis that the neuropsychological presentation of Korsakoff’s syndrome, the chronic phase of Wernicke–Korsakoff syndrome (WKS), is invariably a severe, selective amnesia against a background of relatively preserved general intellectual functions in a consecutive clinical sample. An analysis of the neuropsychological profiles of nine cases with a recorded history of WKS was undertaken. All cases were adult males (ages 32 to 70) with a long history of alcohol use disorder. Eight cases were chosen retrospectively on a consecutive basis from patient referrals. One additional case was recruited prospectively. Conventional understanding and some current opinion of Korsakoff’s syndrome predicts anterograde memory to be consistently more impaired than other cognitive abilities, but this was not found in this case series. The Mean Wechsler Delayed Memory Index was not significantly different from the Wechsler Full-Scale IQ (FSIQ), *p* = 0.130. Regression of Delayed Memory on FSIQ produced a non-significant intercept, *p* = 0.213. The ‘hallmark’ criterion of anterograde memory score at least 20 points less than intelligence score was observed in four of eight cases with available data, equating to a ‘sensitivity’ of 50%. Three of eight cases with available data had an FSIQ less than the memory score. Contrary to a common view, general intellectual function was not consistently preserved in Korsakoff’s syndrome relative to memory function. This study illustrates one of the specific merits of case series, namely, to critique an established view. Clinicians and researchers should expand their diagnostic criteria for Korsakoff’s syndrome to include more variable cognitive phenotypes, including a potentially reversible dementia-like impairment of variable severity, and focus on potential treatment opportunities.

## 1. Introduction

The chronic phase of Wernicke–Korsakoff syndrome (WKS), namely Korsakoff’s syndrome, is conventionally defined as disproportionately impaired anterograde memory relative to other cognitive functions [1,2,3,4]. The DSM-5 makes no mention of cognitive deficits in Korsakoff’s syndrome other than memory impairment and confabulation [5]. A widely accepted psychometric definition of Korsakoff’s syndrome involves a 20–30-point discrepancy between intelligence and anterograde memory quotients [1,6,7].

Although the term ‘Memory Quotient’ (MQ) from the original Wechsler Memory Scale [8] is obsolete, the modern equivalent is the General or Delayed Memory Index from more recent versions of the Wechsler Memory Scales or any similar measure of anterograde or long-term retrieval, memory [9,10]. Due to the obsolescence of the MQ, explicitly characterizing Korsakoff’s syndrome in terms of an IQ–MQ discrepancy is now less common. Nevertheless, as noted, contemporary definitions of Korsakoff’s syndrome continue to suggest that a large discrepancy between anterograde memory ability and general intellectual ability is the clinical hallmark of Korsakoff’s syndrome [1,3,11].

In contrast, it has been acknowledged by many that the acute and chronic phases of WKS are more heterogeneous, both in neurological and cognitive terms, than commonly appreciated. Both acute and chronic WKS can manifest cognitive deficits of varying severity and fluctuating course [12,13,14,15,16]. Also, there is no non-arbitrary distinction between the neuropathology of the Wernicke’s encephalopathy phase and the Korsakoff’s syndrome phase, apart from chronicity or duration of disease [17,18]. In addition, there is no non-arbitrary distinction between the cognitive impairment in the Wernicke’s encephalopathy phase versus the Korsakoff’s syndrome phase [12,16,17,19,20]. As a consequence, it has been suggested that the acute (Wernicke’s encephalopathy) and chronic (Korsakoff’s syndrome) phases should be described under the broader term of WKS, recognizing that WKS has a highly variable course in terms of severity and chronicity [12,16,19,20,21,22].

In this vein, it has been argued that the older, obsolete diagnostic criterion for Korsakoff’s syndrome described above, namely, that memory is disproportionately impaired compared to other cognitive functions, may be an artifact of a research heuristic that has limited diagnostic sensitivity [12,16,20]. Recently, Michael Kopelman (2022) [1] sought to perpetuate the definition of the Korsakoff phase of WKS as strictly conforming to a disproportionate memory impairment by citing, inter alia, the definition of Victor et al., 1971 [23] that predates the most important advance in our understanding of the condition in the last half-century, namely more than 80% of cases of WKS pathology are not diagnosed in life. The revelation that most cases are not correctly diagnosed in life led most writers to radically change their view of the clinical phenotype, abandoning the classic triad definition of Wernicke’s encephalopathy as severely lacking in diagnostic sensitivity and abandoning the severe selective amnesia definition as lacking sensitivity to the Korsakoff phase [16,24,25]. In the words of Reuler et al. (1985) [14], “many alcoholics with chronic cognitive impairment may have had subclinical, unrecognized episodes of Wernicke’s encephalopathy” (p. 1037).

Instead, Kopelman (2022) [1] argues that patients with an alcohol-use disorder and broader chronic cognitive impairment should be considered as suffering from alcohol-related brain damage and cites Lishman’s endorsement of the then popular concept, “Lishman (1986, 1990) wrote of ‘alcoholic dementia’ arising from cortical atrophy secondary to a direct neurotoxic effect of alcohol” ([1], p. 11). But Kopelman does not mention Lishman’s later revised view in light of the post-mortem evidence on undiagnosed WKS, “Nowadays the idea of a genuine dementia caused by alcohol is quite commonly viewed with caution. Many patients labelled as alcoholic dements are indeed suffering from Korsakoff’s syndrome (or another coincident dementia)” (Lishman, 1998 [25], p. 603).

Similarly, Kopelman (2022) [1] repeatedly cites Victor et al. (1971) [23] as endorsing the disproportionate memory impairment definition of the Korsakoff phase but does not cite Victor’s (1994) [16] later view, again in response to the evidence on missed clinical diagnosis, “perhaps this notion of the Korsakoff amnesic syndrome [as severe, selective amnesia], is the conventional one, but if so, it is not consonant with the observed facts, clinical or pathologic ([16], p. 92). “Contrary to common belief, the defects in learning and memory improve slowly, sometimes to a considerable degree” ([16], p. 93). “It is manifestly illogical to designate one phase of this mental disorder by one name (Korsakoff psychosis) and the more chronic phase by another (alcoholic dementia or deteriorated state), as though they were different diseases” ([16], p. 93). In his paper, Kopelman (2022) [1] acknowledges that cognitive impairments other than memory can be affected in WKS to varying degrees and that there are mild or moderate presentations of Korsakoff’s syndrome. However, he concludes that the best way to define Korsakoff’s syndrome is to restrict the definition to the “core feature”, which is a disproportionate memory impairment. However, a review of clinical studies suggests that this definition excludes a substantial proportion, perhaps the majority, of variants of Korsakoff’s syndrome or chronic WKS [12,16,19,20,21], an exclusion that has been overlooked in many scholarly accounts.

The critical component of Kopelman’s (2022) [1] argument for preserving the disproportionate memory impairment definition of the Korsakoff phase of WKS is the view that such a definition is representative and characteristic of patients in the Korsakoff phase. However, this argument is based on highly selected case-series evidence (for review, see Bowden, 1990 [12]), namely, only Level 4 evidence for a diagnostic standard (Oxford Centre for Evidence-Based Medicine OCEBM: https://www.cebm.ox.ac.uk/resources/levels-of-evidence/ocebm-levels-of-evidence (accessed on 17 September 2023) [26]). No consecutive or representative sampling of a clinical cohort with a consistent reference standard (Level 2 evidence: OCEBM) is presented by Kopelman to support his view.

A search of the literature has not located any consecutive case series of patients with chronic WKS in whom detailed cognitive assessment has been reported in a representative series of patients selected on the basis of neurological diagnosis alone, without the addition of the potential biasing effect of the relatively preserved intelligence versus impaired anterograde memory criterion described above (see Bowden, 1990 [12]). Instead, most cognitive studies appear to report on non-representative samples of chronic WKS or Korsakoff’s syndrome. That is, cases that do not conform to a neuropsychological stereotype of severe selective amnesia [6] are typically excluded from the study [12]. However, the diagnostic criterion of severe, selective amnesia was promulgated without any evaluation of diagnostic sensitivity and specificity and in the context of widespread acknowledgment that the criterion excluded many patients with a neurological diagnosis of chronic WKS [6,27]; for reviews, see [12,16]. Indeed, Nelson Butters acknowledged that the IQ–MQ discrepancy was adopted arbitrarily as a clinical definition on the basis of authoritative opinion, without any relevant validation study regarding what we would now term sensitivity and specificity against a credible reference standard such as a post-mortem diagnosis (N. Butters, personal communication to S. Bowden, February, 1993).

The objective of this short, consecutive case series was to evaluate the hypothesis that the neuropsychological presentation of chronic or post-acute WKS is one of severe, selective memory impairment against a background of relatively preserved general intellectual function.

## 2. Materials and Methods

### 2.1. Selection

The case series comprised nine individuals with recorded neurological histories of WKS. Eight cases were identified retrospectively from consecutive referrals to the Neuropsychology Unit, St. Vincent’s Hospital Melbourne, Australia. One additional case was identified prospectively and assessed as an inpatient (Case 1). Retrospective cases had undergone routine neuropsychological assessments as either outpatients or inpatients between 2004 and 2011. Cases were included if they had (1) a recorded medical diagnosis of WKS or any variant, especially Wernicke’s encephalopathy, and (2) completed a detailed neuropsychological assessment from which meaningful comparisons could be made between general intellectual function and anterograde memory function. One case diagnosed with WKS was not included in the study due to insufficient cognitive assessment information. Apart from Case 1, reported below, all cognitive assessments were administered by neuropsychologists not related to the study. Exclusion criteria were any other known cause of cognitive impairment. Three cases were excluded for this reason, specifically due to history of traumatic brain injury, suspected multi-infarct dementia, or “HIV dementia,” respectively.

Eight of the nine cases had a recorded medical file history of at least one acute episode of WKS or Wernicke’s encephalopathy. The other case (Case 9) had no recorded history of acute WKS but was diagnosed with Korsakoff’s syndrome. For six of the eight cases with an acute episode recorded, the acute presentation was diagnosed as Wernicke’s encephalopathy by treating medical staff. For the remaining two cases (Case 2 and Case 6), the acute episode was characterized by confusion and ataxia in the context of alcohol use disorder and poor diet. Although the term Wernicke’s encephalopathy was not recorded for Case 2 and Case 6, both met established criteria for acute Wernicke’s encephalopathy [17,28]. In addition, both Case 2 and Case 6 were diagnosed with WKS by a neuropsychologist not associated with the present study.

All cases were males between 32 and 70 years of age (mean age = 53.33, standard deviation = 12.12) with a history of alcohol use disorder. Table 1 provides summary of demographic and medical history information for each case, including details of estimated alcohol use and most recent recorded episode of acute WKS, sometimes termed Wernicke’s encephalopathy, in the medical files. Neurological signs listed in Table 1 that were recorded during the last acute episode of WKS were not all present at the time of neuropsychological assessment. In particular, no patient was reported to be in an acute confusional state or had a reduced level of consciousness at the time of neuropsychological assessment. That is, all patients remained fully alert and aware of their environment during assessment and were able to attend to tasks throughout the assessment.

### 2.2. Measures

Six different standardized test batteries were used to measure memory and general intellectual function. These are summarized in the Table 2 legend. Dependent variables included all available test index scores, primarily Full-Scale IQ (FSIQ, or general intellectual function) and Delayed Memory Index. Normative data for each test index, as provided in the respective test manuals, show that each index has a mean of 100 and standard deviation of 15 [29].

### 2.3. Ethical Approval

The Human Research Ethics Committee, St. Vincent’s Hospital Melbourne, Australia, granted approval to conduct all elements of this study, including use of prospectively and retrospectively obtained patient data. Written informed consent was obtained for prospectively recruited cases. SVHM HREC reference: 114/11.

### 2.4. Procedure and Statistical Analysis

All data were obtained from the medical and neuropsychology files of each patient except for Case 1, who was assessed by one of the authors (SJS).

At the group level, a two-tailed paired *t*-test was conducted to compare general intellectual ability (FSIQ) and delayed memory ability. To compare general intellectual ability (FSIQ) and delayed memory at the level of the individual, the simple-difference method was used, as described in the Technical and Interpretative Manual of the Wechsler Memory Scale, Fourth Edition [30]. To calculate the critical values required for the simple-difference method, age-based reliability coefficients were obtained from each of the relevant test manuals [29,30,31,32,33].

## 3. Results

Table 2 presents index scores for each case. The mean full-scale IQ (FSIQ) for the sample with available data was 85.67 (*SD* = 16.82; 95% CI = 72.74, 98.59). The mean Delayed Memory Index was 74.13 (*SD* = 20.38; 95% CI = 57.09, 91.16). The discrepancy between FSIQ and Delayed Memory Index indicated the latter was 11.75 index points lower, on average (*SD* = 19.40; 95% CI = −4.47, +27.97). A two-tailed paired-sample *t*-test revealed that the mean Delayed Memory Index score was not significantly different from the mean FSIQ, *t*7 = 1.71, *p* = 0.130, with a standardized mean difference or Cohen’s *d* of 0.61, a small to medium difference. Using Wechsler Scale score-range descriptors, scores in the range of Extremely Low (index score less than 70) to Average (index score between 90–110) were reported for both FSIQ and Delayed Memory Index in the sample of nine participants.

Regression of Delayed Memory on FSIQ produced a non-significant slope coefficient (standardized slope = 0.49; 95% CI = −0.32, 0.89; *p* = 0.213). The confidence interval of the slope, which is wide because of the small sample, includes the same figure reported for the correlation between FSIQ and delayed memory ability in the general population (*r* = 0.61 for FSIQ and Delayed Memory Index; [30]). Therefore, we cannot reject the hypothesis that the relationship between intelligence and anterograde memory ability in this sample of patients with WKS is similar to the population correlation.

The value of the regression intercept also failed to reach significance (intercept = +25.99; 95% CI = −60.22, 112.20; *p* = 0.489). Although an imprecise estimate because of the small sample size, this regression intercept does not support the hypothesis that delayed memory was more impaired than intelligence. The intercept should be significant and negative for the regression of memory on intelligence, as predicted by the commonly held view of severe selective amnesia [5,6]. A summary of the regression analysis is provided in Table 3.

On a case-by-case basis, and as described above, the classic definition of post-acute or chronic WKS would predict significant differences between general intellectual ability (FSIQ) and delayed memory function, always in favor of higher general intellectual ability. Therefore, a large positive difference should be observed when the Delayed Memory Index is subtracted from FSIQ. As shown in the right column in Table 2, the difference between FSIQ and Delayed Memory Index is reported for all cases except Case 8, which lacked data on the indexes. For five of these eight cases (62.5%), the value of the IQ–MQ discrepancy was positive, indicating memory function was poorer than general intellectual function. The discrepancy was statistically significant for all five cases (simple-difference method, *p* < 0.01). For the remaining three cases (37.5%), the value of the IQ–MQ discrepancy was negative, indicating memory function was better than general intellectual function. The discrepancy between FSIQ and the Delayed Memory Index was statistically significant for one of the three cases with negative IQ–MQ discrepancy (simple-difference method, *p* < 0.01).

For Case 8, the neuropsychology report stated that Case 8 was administered an abbreviated memory assessment due to the patient’s “low frustration tolerance”. Therefore, the Delayed Memory Index and FSIQ discrepancy could not be reported for Case 8. Nevertheless, with the use of the respective test manuals, the following was noted for Case 8 [34,35]. As shown in Table 2, Case 8 scored in the Average range for the Verbal Comprehension Index (VCI), Working Memory Index (WMI), and on a measure of auditory–verbal delayed memory (Logical Memory II). Case 8 scored in the Low Average range for the Perceptual Reasoning Index (PRI) and FSIQ. His Processing Speed Index (PSI) was in the Borderline range, and he scored Extremely Low on a measure of visual delayed memory (Visual Reproduction II). Using the critical values supplied in the test manuals, differences between indexes were statistically significant for VCI versus PRI, VCI versus WMI, VCI versus PSI, and PRI versus WMI (*p* < 0.05; [34]). Although an IQ–MQ discrepancy could not be reported for Case 8, his neuropsychological profile indicated reduced function in multiple cognitive domains but relatively preserved verbal memory function (see Table 2).

For the eight cases with a recorded episode of acute WKS, Spearman’s correlations were conducted to investigate potential relationships between the key test indexes and the time between the last known acute episode and neuropsychological assessment. Spearman’s correlation was not significant for any of the relationships between the time (in days) since last acute episode and (1) FSIQ (*r_s_* = −0.18; *n* = 8; *p* = 0.670, two-tailed test), (2) Delayed Memory Index (*r_s_* = +0.22; *n* = 7; *p* = 0.638, two-tailed test), or (3) IQ–MQ discrepancy (*r_s_* = −0.31; *n* = 7; *p* = 0.504, two-tailed test).

## 4. Discussion

Many previous authors have highlighted the cognitive heterogeneity of chronic WKS (for reviews, see [12,16,19,21]). However, it is difficult to find detailed psychometric descriptions of patients with the chronic phase of WKS who do not conform to what has become the predominant criterion for diagnosis of the condition, namely severe, selective amnesia. To address this gap in the literature, the cognitive profiles of nine patients with recorded neurological histories of WKS were analyzed. In this short consecutive case series, the results failed to support the hypothesis that chronic WKS or Korsakoff’s syndrome is consistently characterized by a severe, selective memory impairment. This latter view accords with a review of the historical literature and clinical studies [12,16,20].

Instead, the ‘hallmark’ criterion of delayed memory score at least 20 points less than the intelligence score [6,7] was only observed in four of the eight cases with the available data, equating to a ‘sensitivity’ of 50%. Three cases had an IQ–MQ discrepancy in the opposite direction to that hypothesized (general intellectual ability scoring lower than delayed memory ability), even though the magnitude of the discrepancy was not statistically significant for two of these three cases with index scores available. An additional patient (Case 8) showed no severe, selective memory impairment, although they were assessed with briefer cognitive tests.

Further, if memory function was consistently more impaired than general intellectual function in WKS, then it would be expected that the intercept of the regression of delayed memory on general intellect would be negative and significantly different from zero. Statistical tests showed this not to be the case, although the study with nine cases obviously had low design power. Statistical significance aside, the observed intercept was less than one standard error different from zero and in the wrong direction to that hypothesized by the severe selective amnesia criterion for Korsakoff’s syndrome (see Table 3). Therefore, taken together with the results at the individual level, the regression analysis provides no evidence to support the hypothesis of memory being consistently more impaired than other cognitive abilities. These regression results support the results obtained by Jacobson and Lishman’s (1987) [36] less formal analysis and the regression analysis of Bowden and Ritter (2005) [37] in a different sample, all of whom conformed to the Caine et al. (1997) [17] criteria for WE or WKS, although fewer of the patients in Bowden and Ritter’s study had a known recent acute episode or hospitalization for severe WKS. Cognitive impairment in WKS is variable and more generalized than the established focus on selective memory impairment suggests [1,2,3,11]. Other cognitive presentations are common, including dementia-like and other cognitive variants [12,16,20].

Haalboom et al. (2019) [38] also found that FSIQ is not preserved in chronic WKS. In their study, FSIQ was significantly lower in the group of 34 inpatients with Korsakoff’s syndrome compared to the control group of patients with other psychiatric disorders, but not when compared to patients with an alcohol use disorder and cognitive deficits who did not meet DSM-5 criteria for Korsakoff’s syndrome (i.e., met criteria for Minor Neurocognitive Disorder due to Alcohol [5]). Perceptual Reasoning Index (PRI) and Processing Speed Index (PSI) from the Wechsler Adult Intelligence Scale^®^—Fourth Edition (WAIS^®^—IV) were also significantly poorer in the group with Korsakoff’s syndrome compared to the non-alcoholic control group but again not when compared to the group with alcohol use disorder but no Korsakoff’s syndrome. The authors concluded that different aspects of intelligence are affected differently in chronic WKS, such as fluid intelligence (Gf) being more affected than crystallized intelligence (Gc).

Whether specific aspects of intelligence are relatively preserved and others more vulnerable in chronic WKS warrants further investigation. As it stands, and considering intellectual function in broader terms (e.g., full-scale IQ or FSIQ) as intelligence is treated in contemporary definitions of chronic WKS or Korsakoff’s syndrome, the evidence is mounting that IQ or general intellectual function is also affected in chronic WKS.

Ironically, it is now widely accepted that the neurological manifestations of the Wernicke’s encephalopathy phase of WKS are highly variable, with many cases showing no obvious neurological or cognitive signs, a presumptive diagnosis often being made on the basis of risk factors alone [3,14,17,39]. If it is the case that the Wernicke’s phase of the disease is highly variable, it is illogical to assume that the Korsakoff phase will not be highly variable in clinical manifestations either. This point was made many years ago by Torvik (1991) [20] on the basis of careful observation of the variable pattern and severity of neuropathology in cases of WKS diagnosed at post-mortem.

While eight of the nine cases in the current series had previously experienced a known acute episode of WKS (Wernicke’s encephalopathy), the acute symptoms of WKS were not the reason for the finding of variable or generalized cognitive impairment. A confusional state and reduced level of consciousness are considered features of the acute phase of WKS [3,22]. For the purposes of the present study, it is important to note that all patients underwent neuropsychological assessment at a time when any confusion or reduced level of consciousness had fully resolved, as determined by the neuropsychology reports and patient observation, which indicated that all patients remained fully alert and aware of their environs during assessment and were able to attend to tasks. Also, as noted above, there was no significant correlation between the time since an acute episode and the severity of cognitive impairment (see Table 1). Thus, the objectively observed cognitive deficits cannot be attributed to the acute, confusional phase of WKS.

Even though statistical analysis failed to support a relationship between time since an acute episode of WKS and neuropsychological results, given the small sample size of the study, it is worth noting that five of the nine presented cases completed neuropsychological assessment less than a month after experiencing an acute episode of WKS. Therefore, while the cognitive scores are not attributable to an acute confusional state or lowered level of consciousness, it cannot be ruled out that the time between an acute episode and neuropsychological assessment explains some of the cognitive variability observed in this case series. Such a limitation applies to all historical case reports. The variability in cognitive profiles observed in our representative case series indicates that the conventional diagnostic description of chronic WKS as a disorder characterized by memory disproportionately impaired relative to other cognitive abilities lacks sensitivity, failing to identify a substantial subset of patients with evolving WKS. For example, it would be unreasonable to diagnose a patient experiencing an acute confusional state with acute WKS, then consider the WKS as resolved upon resolution of the acute neurological signs, only to re-diagnose the same patient at any time subsequently with chronic KS because the patient’s memory impairment became the predominant feature of their condition as other cognitive abilities resolved to some extent. To be clear, the reader is reminded that many patients with chronic WKS exhibit significant recovery over time [12,16,22,40], and so, even patients with a classic presentation of chronic WKS at some point may no longer meet traditional criteria after further, partial, or complete recovery.

Aside from memory impairment and so-called executive dysfunction, more pervasive cognitive impairment in WKS is frequently overlooked [12,16,20,21]. Cognitive heterogeneity in chronic WKS has previously been reported in group studies (e.g., Haalboom et al., 2019 [38]) but is greatly underappreciated, even in contemporary accounts [1,2,3,41]. The idea of chronic WKS as a disorder of memory and relatively intact general intellectual function is an enduring consequence of the practice of excluding from published studies any individual who did not conform to the neuropsychological stereotype of chronic WKS [12,16]. The findings of the present case series, as well as the findings of Jacobson and Lishman (1987) [36], Bowden and Ritter (2005) [37], and Haalboom et al., 2019 [38]) are not consistent with the established definition of WKS [2,4,5,6]. Also frequently overlooked is the scope for recovery in the Korsakoff’s syndrome phase of WKS [12,16,22,40].

### 4.1. Study Limitations

The small sample size and associated lack of statistical power is a limitation to be considered, primarily when interpreting non-significant statistical findings. However, as alluded to above, statistics aside, a review of the neuropsychological assessment results for each individual case (see Table 2) is a sufficient demonstration of the poor sensitivity of the conventional definition of chronic WKS as severe selective amnesia.

Another limitation of the present case series was the possibility of unknown extraneous causes of cognitive impairment, such as unreported head injury, polysubstance abuse, liver disease, hepatic encephalopathy, pancreatitis, and multiple organ disease. Case 3 was suspected of having experienced a hypoxic episode of unknown severity, although there was no reason to infer significant cognitive sequelae in that case. Extraneous causes of cognitive impairment may have attenuated cognitive and neurological function to some extent, but it is a ubiquitous feature of patients with alcohol-use disorders and does not weaken the above finding of no consistent pattern of relative impairment in comparison to general intellectual function (FSIQ) and memory in WKS. Complicating illnesses are common in people with severe alcohol use disorder, and these complications are likely to have confounded most published studies of patients with WKS to some extent, including those studies that confidently describe severe selective amnesia as pathognomonic. In the above WKS sample, general intellectual function was not consistently preserved relative to memory function; rather, most cases were judged to suffer some decline in general intellectual function in addition to memory impairment.

### 4.2. Alcohol-Related Dementia: A Problematic Diagnosis

The improved recognition of variability in the clinical presentation of chronic WKS may help to explain the common observation of alcohol-related dementia, a diagnostic entity that has always been controversial. Instead, alcohol-related dementia has been attributed to variants of WKS (for reviews, see [12,15,16,19,25]). The prevalence of WKS neuropathology in patients with a history of alcohol use disorders is estimated at approximately 12–35% [20,42], which coincides with the estimates of dementia in patients with alcohol use disorders 9–25% (for a review, see Ritchie and Villebrun, 2008 [43]). In addition, it is widely acknowledged that alcohol-related dementia lacks a pathological gold standard, and the most common neuropathology underlying clinical dementia in patients with alcohol use disorders is WKS [11,16,17,24,44]. As Torvik and colleagues noted many years ago, once cases of Alzheimer’s disease and other “coincidental conditions” are excluded, “all alcoholics that have been labelled demented will turn out to have inactive [chronic] Wernicke’s encephalopathy” ([15], p. 245).

Regardless of diagnosis, prophylactic treatment or restoration of thiamine levels should be considered in any patient presenting with cognitive deficits in the context of an alcohol use disorder [14]. Accurate diagnostic labeling will also lead to better psychoeducation and counseling for the patient and their family. For example, a patient diagnosed with “alcohol-related dementia” may conflate their knowledge of their condition with their pre-existing understanding of neurodegenerative dementias. The patient, family, and clinician may also fail to appreciate the scope for recovery when the wrong label is used [12,16,22]. Accurate diagnostic labeling and better psychoeducation will also help all to highlight the importance of adequate nutrition and the impact of alcohol on the patient’s nutritional status, especially with respect to thiamine or Vitamin B1.

## 5. Conclusions

In conclusion, variability in objective psychometric profiles was evident in cases of WKS recruited on the basis of neurological signs and medical presentation alone without potentially biasing effects of a preconceived psychometric profile. These results provide Level 2 diagnostic evidence (OCEBM) [26] and support the view that WKS produces a spectrum of cognitive impairment [12,16,19,20]. Davison and Lazarus (2007) [45] highlighted the well-known view that uncontrolled case studies (Level 4 evidence) have limitations in terms of generalization. Nevertheless, these authors highlight one of the distinct benefits of case studies if providing information that contradicts a prevailing view of the respective disorder. In this study, the observation of a proportion of patients with chronic WKS with a cognitive profile that does not conform to the prevailing view provides compelling evidence that the prevailing view is not accurate. It is recommended that clinicians and researchers expand their diagnostic or patient selection criteria for chronic WKS to include more variable cognitive profiles. Enhanced understanding and recognition of the heterogeneity and different subtypes of WKS could lead to better detection of this often-undiagnosed disease and, hence, better treatment [19,46].

## Figures and Tables

**Table 1:** Patient background information and summary of most recent acute episode of Wernicke–Korsakoff Syndrome (WKS).

Case Number	Age-Gender	Marital Status	Details of Alcohol Use *	Other Risk Factors for Nutritional Deficiency	Time between Acute Episode of WKS and Neuro-Psychological Assessment	Details of Most Recent Acute Episode					Additional Information
						Number of Signs of Classic Triad **	Mental Signs ***	Eye Signs	Ataxia	Neuro- Imaging	
1	51 m	Divorced	>20 years approx. 85–341 g/day	None known	7 days	3	Confusion, reduced conscious state	Nystagmus ophthalm-oplegia	Ataxic gait	CT-Small old lacunar infarct. MRI-Mild global atrophy	-
2	39 m	Never married	>20 years approx. 363–400 g/day	Minimal eating, diarrhea, oesophagitis, gastritis	<17 days	2	Confusion	-	Limb and truncal ataxia	CT-Global atrophy, no focal lesion	Chronic liver disease, hepatic encephalopathy, withdrawal seizures
3	32 m	Never married	15 years approx. 200–300 g/day	Minimal eating, dysphagia, mild gastritis	2–3 weeks	3	Confusion, reduced conscious state, confabulation, drowsiness	Nystagmus	Ataxia	CT and MRI-Generalized cerebral atrophy, no acute abnormality	Chronic liver disease, hepatic encephalopathy, polysubstance abuse, hypoxic event
4	64 m	Separated	8–20 years approx. 363–400 g/day	Vomiting secondary to chronic reflux, swallowing difficulties	5–6 months	3	Confusion, altered conscious state, confabulation	Ophthalm-oplegia	Ataxia	CT-Normal	History of psychosis
5	62 m	Divorced	- approx. 146–195 g/day	Vomiting, diarrhea, loss of appetite	14 days	2	Acute confusional state, lethargy	Diplopia (3 years), nystagmus, ophthalm-oplegia	-	CT-Normal	Questionable past CVA/TIA
6	49 m	Never married	Not available	Food refusal in days preceding admission, oesophagitis	<14 days	2	Confusion	-	Ataxic gait	CT-Normal	Chronic liver disease
7	58 m	Widowed	Not available	Minimal eating, chronic abdominal pain, oesophagitis, gastritis	1 month	2	Confusion, reduced conscious state, confabulation	-	Ataxic gait	CT-Normal MRI-Normal	Pancreatitis, heart disease, COPD, seizure
8	55 m	Married	36–40 years approx. 136–180 g/day	Minimal eating, weight loss	6–7 months	3	Confusion, lethargy	Nystagmus	Ataxic gait	CT-Cerebral atrophy, predominantly in frontal lobes	Chronic liver disease, peripheral neuropathy, chronic pancreatitis, COPD, withdrawal seizure
9	70 m	Married	Details of past abuse not available Current: approx. 20–30 g/day	Barrett’s oesophagus, hiatus hernia	No history of acute episode					MRI-Hyperintensity in right pons resolved at time of assessment	Peripheral neuropathy, sleep apnea

* g/day corresponds to approximate estimate of grams of ethanol consumed per day via alcoholic beverages. ** The classic clinical triad of neurological signs of acute WKS (Wernicke’s encephalopathy) are mental signs (lowered level of consciousness, including confusion and coma, and reduced cognitive functioning), eye signs (nystagmus, ophthalmoplegia), and ataxia. *** Any confusion or reduced level of consciousness had resolved by time of neuropsychological assessment. Abbreviations: CVA—cerebrovascular accident; TIA—Transient ischemic attack; COPD—Chronic obstructive pulmonary disease.

**Table 2:** Summary of neuropsychological assessment scores derived from respective versions of Wechsler Intelligence or Memory Scales (see footnotes for details).

Case Number		VCI	POI/PRI	WMI/Attention	PSI	VIQ	PIQ	FSIQ	Immed Mem	Delay Mem	IQ–MQ
1 ^a^	Index score	114	80	102	65			94	77	58	36 *p* < 0.01
	95% CI	108–119	84–97	95–109	60–77	-	-	90–98	72–84	54–67	
	Percentile	82	25	55	1			34	6	0.3	
2 ^b^	Index score	84	86		73	82	78	78	84	92	−14 *p* < 0.01
	95% CI	79–90	80–94	-	67–85	78–87	73–86	74–83	77–93	85–101	
	Percentile	14	18		4	12	7	7	14	30	
3 ^b^	Index score	103	76	80	69	95	70	83	91	92	−9 *p* > 0.05
	95% CI	97–109	70–85	74–88	64–82	90–100	65–79	79–87	84–100	85–101	
	Percentile	58	5	9	2	37	2	13	27	30	
4 ^b^	Index score	84	78	88	69	90	73	80	45	49	31 *p* < 0.01
	95% CI	79–90	72–87	82–95	64–82	85–95	68–81	76–84	42–58	46–62	
	Percentile	14	7	21	2	25	4	9	<0.1	<0.1	
5 ^c^	Index score			91		64	69	64	78	48	16 *p* < 0.01
	95% CI	-	-	79–103	-	60–71	65–76	61–69	67–89	37–59	
	Percentile			27		1	2	1	7	<0.1	
6 ^b^	Index score	112	84			110	78	95	74	75	20 *p* < 0.01
	95% CI	106–117	78–92	-	-	105–115	73–86	91–99	68–84	69–85	
	Percentile	79	14			75	7	37	4	5	
7 ^b^	Index score	78	74	80		78	68	71	73	79	−8 *p* > 0.05
	95% CI	73–85	69–83	74–88	-	74–84	63–77	68–76	67–83	73–89	
	Percentile	7	4	9		7	2	3	4	8	
8 ^a^	Index score	103	81	92	71			84	Logical Memory II age scaled score: 11 Visual Reproduction II age scaled score: 1		n/a
	95% CI	97–109	76–88	86–99	66–82	-	-	80–88			
	Percentile	58	10	30	3			14			
9 ^b^	Index score	136	109	121	96	134	105	122	91	100	22 *p* < 0.01
	95% CI	129–140	101–116	113–127	88–105	128–138	98–111	117–126	84–100	92–108	
	Percentile	99	73	92	39	99	63	93	27	50	

Tests administered. ^a^ Wechsler Adult Intelligence Scale^®^—Fourth Edition (WAIS^®^-IV) and Wechsler Memory Scale^®^—Fourth Edition (WMS^®^-IV). ^b^ Wechsler Adult Intelligence Scale^®^—Third Edition (WAIS^®^-III) and Wechsler Memory Scale^®^—Third Edition (WMS^®^-III). ^c^ Wechsler Abbreviated Scale of Intelligence^®^ (WASI^®^) and Repeatable Battery for the Assessment of Neuropsychological Status (RBANS^TM^). Abbreviations: 95% CI—95% Confidence Interval; VIQ—Verbal IQ; PIQ—Performance IQ; FSIQ—Full-Scale IQ; VCI—Verbal Comprehension Index; POI—Perceptual Organization Index; PRI—Perceptual Reasoning Index; WMI—Working Memory Index; PSI—Processing Speed Index; Immed Mem—Immediate Memory Index; Delay Mem—General or Delayed Memory Index; IQ–MQ—discrepancy when General or Delayed Memory Index score subtracted from FSIQ. Statistical analysis. *p* < 0.01 Difference between FSIQ and Delayed Memory Index score was statistically significant (simple-difference method, *p* < 0.01). *p* > 0.05 Difference between FSIQ and Delayed Memory Index score was not statistically significant (simple-difference method, *p* > 0.05). n/a—Not applicable. IQ–MQ could not be calculated because Delayed Memory Index score was not available for Case 8. According to the neuropsychology report, memory assessment was abbreviated due to the patient’s “low frustration tolerance”.

**Table 3:** Summary of regression analysis predicting Delayed Memory Index on the Wechsler Memory Scale from Wechsler Full-Scale IQ (*n* = 8).

Model Summary							
		Unstandardized regression coefficients		Standardized coefficients	*R* square		
	*B*	95% Confidence Interval	Standard error	Beta			
(Constant)	25.99	[−60.22, 112.20]	35.23				
FSIQ	0.56	[−0.43, 1.55]	0.40	0.49	0.24	*F*(1,6) = 1.94	*p* = 0.213

Predictors: (Constant), Full-Scale IQ (FSIQ, general intellectual function). Dependent Variable: Delayed Memory.

## Data Availability

Data are contained within the article and the cited test manuals.
